# Early Cambrian Pentamerous Cubozoan Embryos from South China

**DOI:** 10.1371/journal.pone.0070741

**Published:** 2013-08-12

**Authors:** Jian Han, Shin Kubota, Guoxiang Li, Xiaoyong Yao, Xiaoguang Yang, Degan Shu, Yong Li, Shunichi Kinoshita, Osamu Sasaki, Tsuyoshi Komiya, Gang Yan

**Affiliations:** 1 Early Life Institute and Department of Geology and State Key Laboratory of Continental Dynamics, Northwest University, Xi'an, P.R. China; 2 Seto Marine Biological Laboratory, Field Science Education and Research Center, Kyoto University, Shirahama, Nishimuro, Wakayama, Japan; 3 State Key Laboratory of Palaeobiology and Stratigraphy, Nanjing Institute of Geology and Palaeontology, Chinese Academy of Sciences, Nanjing, China; 4 School of Earth Science and Land Resources, Key Laboratory of Western China's Mineral Resources and Geological Engineering, Ministry of Education, Chang'an University, Xi'an, P.R. China; 5 Tohoku University Museum, Tohoku University, Aramaki, Aoba-ku, Sendai, Japan; 6 Department of Earth Science and Astronomy, Graduated School of Arts and Science, Tokyo University, Meguro-ku, Tokyo, Japan; 7 Key Laboratory of Unconventional Oil and Gas, China National Petroleum Corporation (CNPC), Langfang, P. R. China; Ecole Normale Supérieure de Lyon, France

## Abstract

**Background:**

Extant cubozoans are voracious predators characterized by their square shape, four evenly spaced outstretched tentacles and well-developed eyes. A few cubozoan fossils are known from the Middle Cambrian Marjum Formation of Utah and the well-known Carboniferous Mazon Creek Formation of Illinois. Undisputed cubozoan fossils were previously unknown from the early Cambrian; by that time probably all representatives of the living marine phyla, especially those of basal animals, should have evolved.

**Methods:**

Microscopic fossils were recovered from a phosphatic limestone in the Lower Cambrian Kuanchuanpu Formation of South China using traditional acetic-acid maceration. Seven of the pre-hatched pentamerous cubozoan embryos, each of which bears five pairs of subumbrellar tentacle buds, were analyzed in detail through computed microtomography (Micro-CT) and scanning electron microscopy (SEM) without coating.

**Results:**

The figured microscopic fossils are unequivocal pre-hatching embryos based on their spherical fertilization envelope and the enclosed soft-tissue that has preserved key anatomical features arranged in perfect pentaradial symmetry, allowing detailed comparison with modern cnidarians, especially medusozoans. A combination of features, such as the claustrum, gonad-lamella, suspensorium and velarium suspended by the frenula, occur exclusively in the gastrovascular system of extant cubozoans, indicating a cubozoan affinity for these fossils. Additionally, the interior anatomy of these embryonic cubozoan fossils unprecedentedly exhibits the development of many new septum-derived lamellae and well-partitioned gastric pockets unknown in living cubozoans, implying that ancestral cubozoans had already evolved highly specialized structures displaying unexpected complexity at the dawn of the Cambrian. The well-developed endodermic lamellae and gastric pockets developed in the late embryonic stages of these cubozoan fossils are comparable with extant pelagic juvenile cubomedusae rather than sessile cubopolyps, whcih indicates a direct development in these fossil taxa, lacking characteristic stages of a typical cnidarian metagenesis such as planktonic planula and sessile polyps.

## Introduction

**Figure 1:**
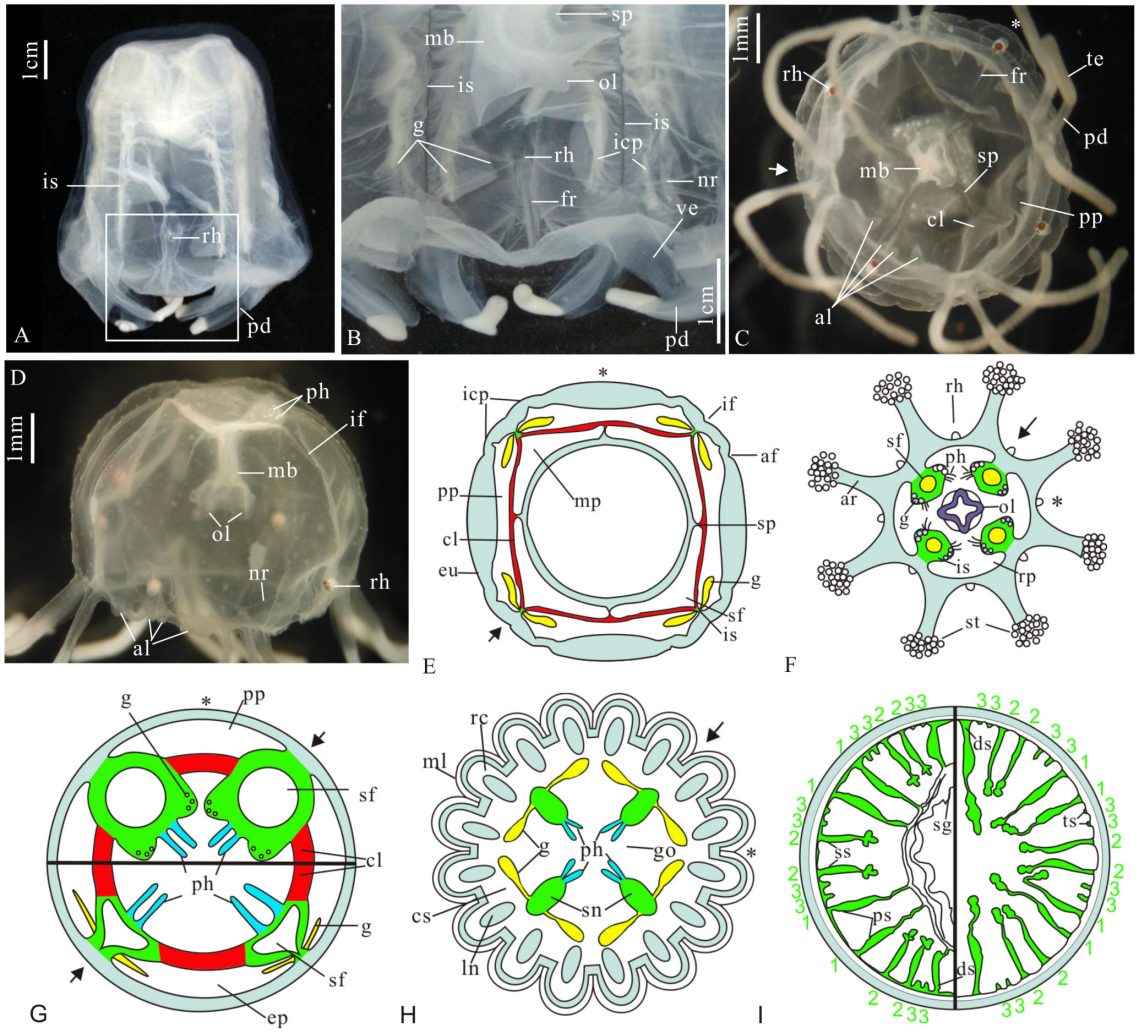
The basic morphology and internal anatomy of living cnidarians (anthozans, staurozoans, coronate scyphozoans, and cubozoans, excepting hydrozoans). (A) A fully grown individual of *Morbakka virulenta* (Kishinouyea, 1910) (Charybdeida, Cubozoa) from Japan, lateral view. (B) Rectangle area in (A), showing the blade-like pedalia, velarium, frenula, interradial septa and gonad-lamellae. (C–D) A juvenile individual of living *Tripedalia cystophora* Conant, 1897 (Charybdeida, Cubozoa) from Japan. (C) Oral view, showing the triangular apertural lappets, four pairs in the perradii and eight in the adradii; note the incipient pedalia. (D) Lateral view. (E) Diagrammatic horizontal section of *Tripedalia cystophora*, showing the internal anatomy, especially the gonad-lamellae and the suspensia, modified from ([17], Figure 23). (F) An oral view of a staurozoans. (G) Diagrammatic comparison of the gonads and gonad-lamellae in horizontal sections between a cubozoan (lower part) and a staurozoan (upper part), modified from ([3], Figures 2–3). (H) Cross section of a coronate scyphozoan showing scalloped bell margin and internal gonads (simplified from Thiel,1966 [3]). (I) Cross section of an ideal hexanerous actiniarian through the pharynx (left) and below the pharynx (right), the numbers indicate the order of insertion of the new septa following the directive septa, modified from [2].

The phylum Cnidaria is a diverse group of relatively simple diploblastic animals with highly complex and typical venomous cell, ‘cnida’. Compared with sessile bilateral polypoid anthozoans, the sub-phylum Medusozoa is characterized by a diagnostic tetra-radial symmetry and a life history including motile planulae, sessile or creeping polyps, and swimming medusae stages. Medusozoans comprise four classes: Staurozoa, Scyophozoa, Hydrozoa and Cubozoa [1]. The latter, popularly known as ‘box jellyfish’ or ‘sea wasps’, is a monophyletic group characterized by a four-sided box-shaped appearance, four bunches of interradial tentacles each with a wing-like pedalium at the proximal end, and four well-developed complex eyes as well as a circular velarium suspended perpendicularly by four bracket-like perradial frenula [1], [2] (see also Figure 1A–B). Internally, the cubomedusa has four interradial septa (mesenteries), four well-developed claustra and four interradial pairs of leaf-like gonads projected into the perradial pockets (or pouches) [3] (see also Figure 1C). Although the systematic position of the Cubozoa is still in debate, it is generally envisioned phylogenetically as a sister group of the Scyphozoa [4]–[6]. Like other medusozoans, the fossil record of cubozoans is quite sparse [7], [8]; a few probable cubozoan fossils with simple and unbranched pedalia are known from the Pennsylvanian Mazon Creek Formation of central USA and the Middle Cambrian Marjum Formation of Utah, western USA [8].

During recent years, a variety of abundant diagenetically phosphatized microscopic fossils and embryos have been documented from the earliest rocks of the Cambrian, in the Kuanchuanpu Formation in Shaanxi Province, southern China (equivalent to the Fortunian Stage, Terreneuvian Series: 541–529 Ma). These fossils include a kind of scyphopolyp with many filiform tentacles [9], some sea anemone-like cnidarians [10], as well as many pentamerous embryos [11], [12]. The latter, referred to as co-occurring *Punctatus emeiensis* and *Olivooides multisulcatus*, have been intimated as having a problematic affinity with coronate scyphozoans [11], [12]. Most recently, three extraordinary embryonic specimens related to *Olivooides* with distinct pentaradial symmetry were reported from the Kucnchuanpu Formation, including a budding ephyra and a specimen with well preserved internal anatomical structures. The latter reveals a double-wall system with radial lobes, radial canals, recurved walls and possible manubrium, but lacking of typical features of echinoderms such as calcite skeleton, vasculae system and a through gut. Thus, these fossils suggest greater compatibility with a cnidarian body plan [13].

**Figure 2:**
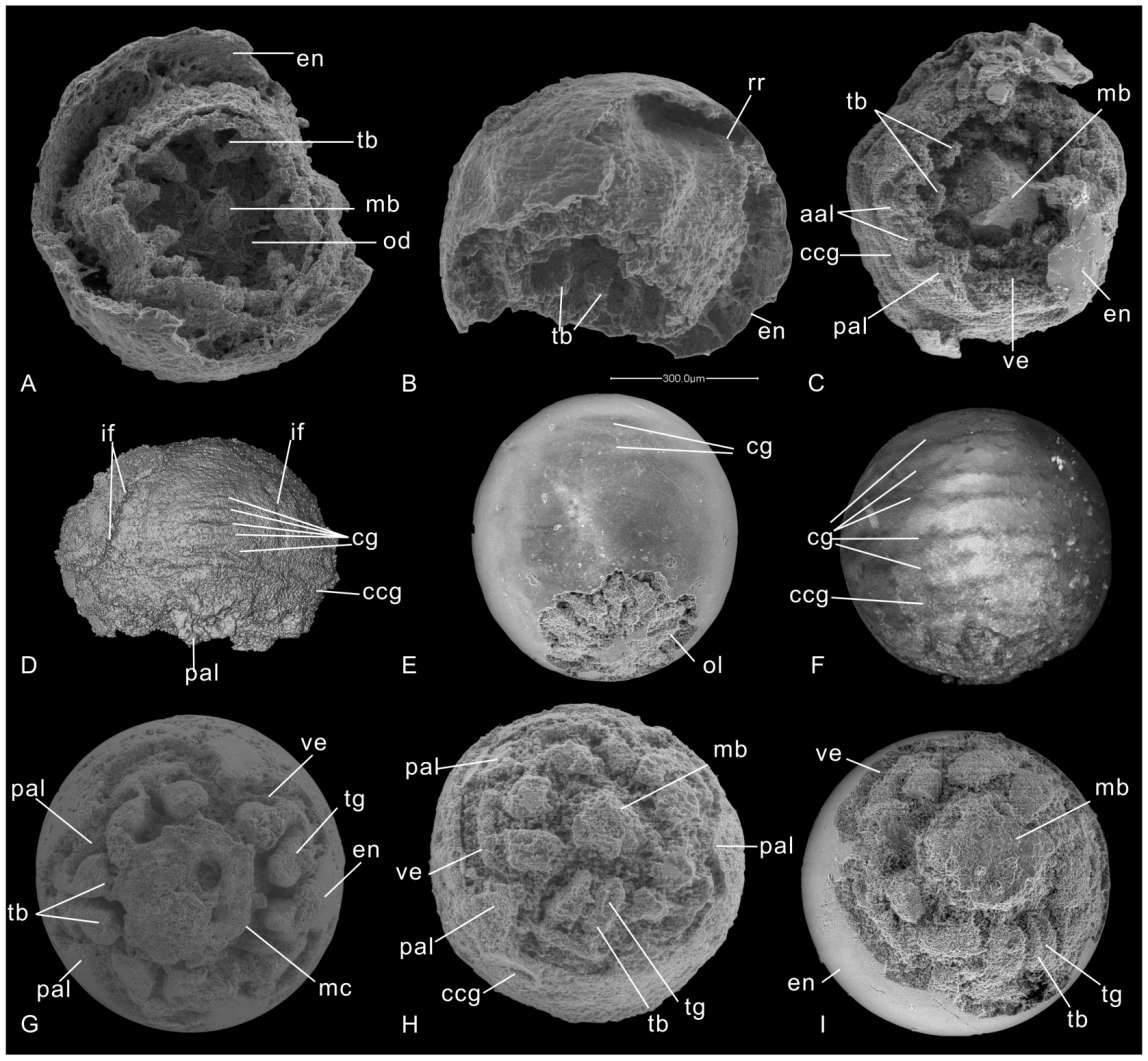
Cubozoan embryos from the Lower Cambrian Kuanchuanpu Formation, Shaanxi, South China. (A–B) SEM photographs of ELISN31-5, oral (A) and lateral view (B) of the SEM photos. (C–D) ELISN108-343; (C) SEM photograph of oral view. (D) Reconstruction of ELISN108-343 from Micro-CT analysis, in lateral view. (E–F) ELISN96-103. (E) SEM photograph, near-lateral view. (F) Lateral view of ELISN96-103 from Micro-CT analysis. (G) SEM photograph of ELISN66-15. (H) ELISN25-79. (I) ELISN35-42. All sections share the same scale bar (equals 300 µm) seen in (B).

In the current research, seven new pentamerous embryos specimens (ELISN31-5, ELISN108-343, ELISN-96-103, ELISN66-14, ELISN66-15, ELISN25-79, ELISN35-42), characterized by five interradial pairs of tentacles surrounding a central manubrium (Figure 2A–I), are found distinct from others previously reported. Specifically, specimens ELISN31-5 and ELISN108-343 have preserved a dazzling array of endodermic lamellae and gastric pockets that are mostly comparable to those of cubozoans rather than coronate scyphozoans. These preserved endodermic layers appear to be directly replaced by microcrystalline apatite and without intervening carbonate mineralization, as previously suggested [11], [14].

## Terminology

The morphological terminology in this paper is in general agreement with previous papers [2], [3], [15], [16]. We introduce here some new terms to define the internal structures of these fossils unknown in extant medusozoans. Abbreviations of the morphological terms in all figures are listed as follows: ?al, ?apertural lappet; aal, adradial apertural lappets; afr, adradial frenulum; af, adradial furrow; aigp, adradial intra-gonad pocket; apcp, adradial peri-claustral pocket; ar, arm; as, accessory septum; bsp, basal septal pocket; ccg, coronal circumferential groove; ccl, cockscomb-lamella; cg, coronal groove; cl, claustrum; clp, claustral projections; cs, coronal stomach; csc, central stomach cavity; ds, directive septum; dwpf, double-walled pentagonal funnel; en, egg envelope; ep, exogonial pocket; es, esophagus; eu, exumbrella; fepf, first endodermic pentagonal funnel; fr, frenulum; g, gonad; gc, gastric cavity; gl, gonad-lamella; go, gastric ostium; icp, interradial corner pillar; if, interradial furrow; igl, inner gonad-lamella; ip, interradial pocket; ipep, interradial peri-esophageal pocket; is, interradial septa; lbl, lower beak-lamella; ln, lappet node; map, marginal adradial pocket; mb, manubrium; mc, manubrial corner; mcg, middle circumferential groove; ml, marginal lappet; mp, mesogonial pockets; mr, manubrial ridge; nl, neck-lamellae; nr, nerve ring; od, oral disc; ogl, outer gonad-lamella; ol, oral lips; pal, perradial apertural lappet; pd, pedalia; pep, peri-esophageal pocket; pgp, peri-gonad pocket; ph, phacellus(gastric filament); pigp, perradial intra-gonad pocket; pmp, perradial mesogonial pockets; pp, perradial pocket; ppcp, perradial peri-claustral pocket; ppep, perradial peri-esophageal pocket; ps, primary septa; rc, radial canal; rh, rhopaloids; rm, retractor muscle; rp, radial pocket; rr, radial ridge; scg, sub-apical circumferential groove; sepf, second endodermic pentagonal funnel; sf, septal funnel; sg, siphonoglyph; sn, septal nodes; sp, suspensorium; sph, stalk of phacellus; srt, septal roots of tentacles; ss, secondary septa; ssp, secondary suspensorium; st, secondary tentacles; su, subumbrella; sve, secondary velarium; svt, secondary velarial teeth; tb, tentacular bud; te, tentacle; tepf, third endodermic pentagonal funnel; tg, tentacle groove; ts, tertiary septa; ubl, upper beak-lamella; vc, velarial canal; ve, velarium; *, perradius; +, adradius; →, interradius.

## Materials and Methods

Fossil preparation and methods of the use of Scanning Electron Microscope (SEM) and Microcomputed tomography (Micro-CT) analysis follow Han et al. [10]. Hundreds of microscopic fossils were examined using Synchrotron radiation X-ray tomographic microscopy (SRXTM) in Spring-8 in Japan. Micro-CT data as well as supplemental movies of figured materials were acquired by Micro-XCT-400 of X-strata. The Micro-CT data can be used to make virtual sections without literally crack the fossils. The data were processed and bundled into an image stack using VG Studio 2.2 that allows detail analysis of these microscopic fossils. Major components in virtual sections of the living and fossil medusae were painted in different colors by using Photoshop 7.0, including interradial septa (red), ph (sky blue), claustra and claustral projections (red), gonad-lamellae (yellow) and accessory septa (pink). All specimens were collected by the first author and have been donated to the Early Life Institute, Northwest University, China (ELI) and deposited there. We have obtained permission from theELI to have free access to the collections.

## Results

### The internal anatomy in specimen ELISN31-5

SEM reveals that the specimen ELISN31-5 is enclosed within a smooth-surfaced fertilization envelope, which was manually partly removed. The enclosed embryo is nearly hemi-spherical, ca. 520 µm in maximal diameter, showing five convex radial ridges (rr) intercalated with five radial depressions at their aboral part (Figure 2A–B),. In the oral view, five evenly spaced pairs of radial lobes, which are rooted at the middle level of the subumbrella and directed horizontally toward the oral-aboral axis, are interpreted here as tentacular buds (tb) or primordial tentacles (Figure 2A–B). The manubrial primordium, with a mouth-pore still closed, is a small, low, cone-like shaft situated below the level of the tentacular buds. Thus superficially the specimen looks like an embryonic medusa.

**Figure 3:**
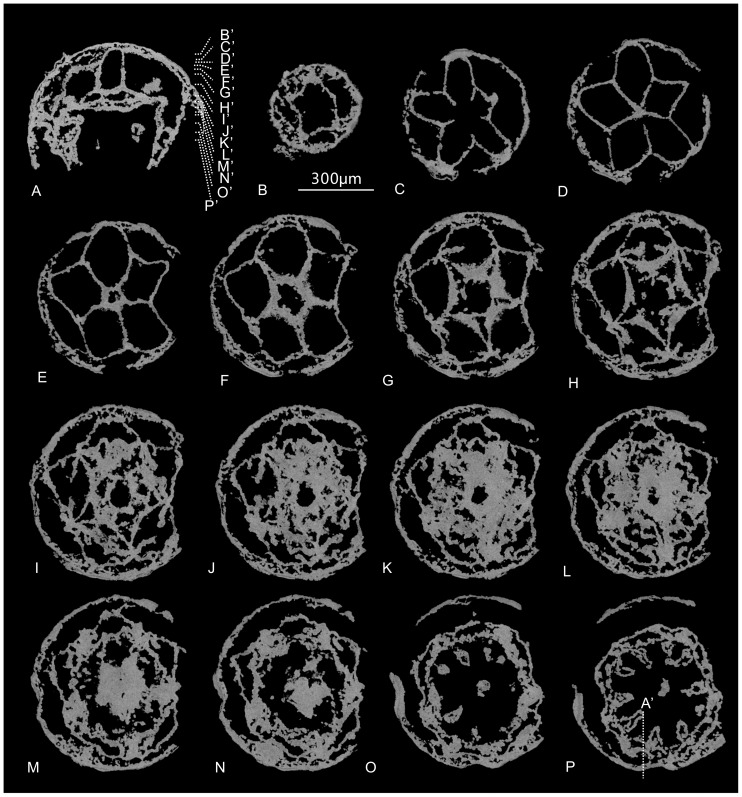
Micro-CT photographs of microscopic cubozoan ELISN31-5 from the Lower Cambrian Kuanchuanpu Formation, South China. (A) Virtual vertical section of ELISN31-5 marked with a vertical dotted line by P′ in (P). (B–P) successive virtual horizontal sections of the same specimen from the aboral pole downward; their horizontal levels are marked by B′–P′ in (A) with white dotted lines.

**Figure 4:**
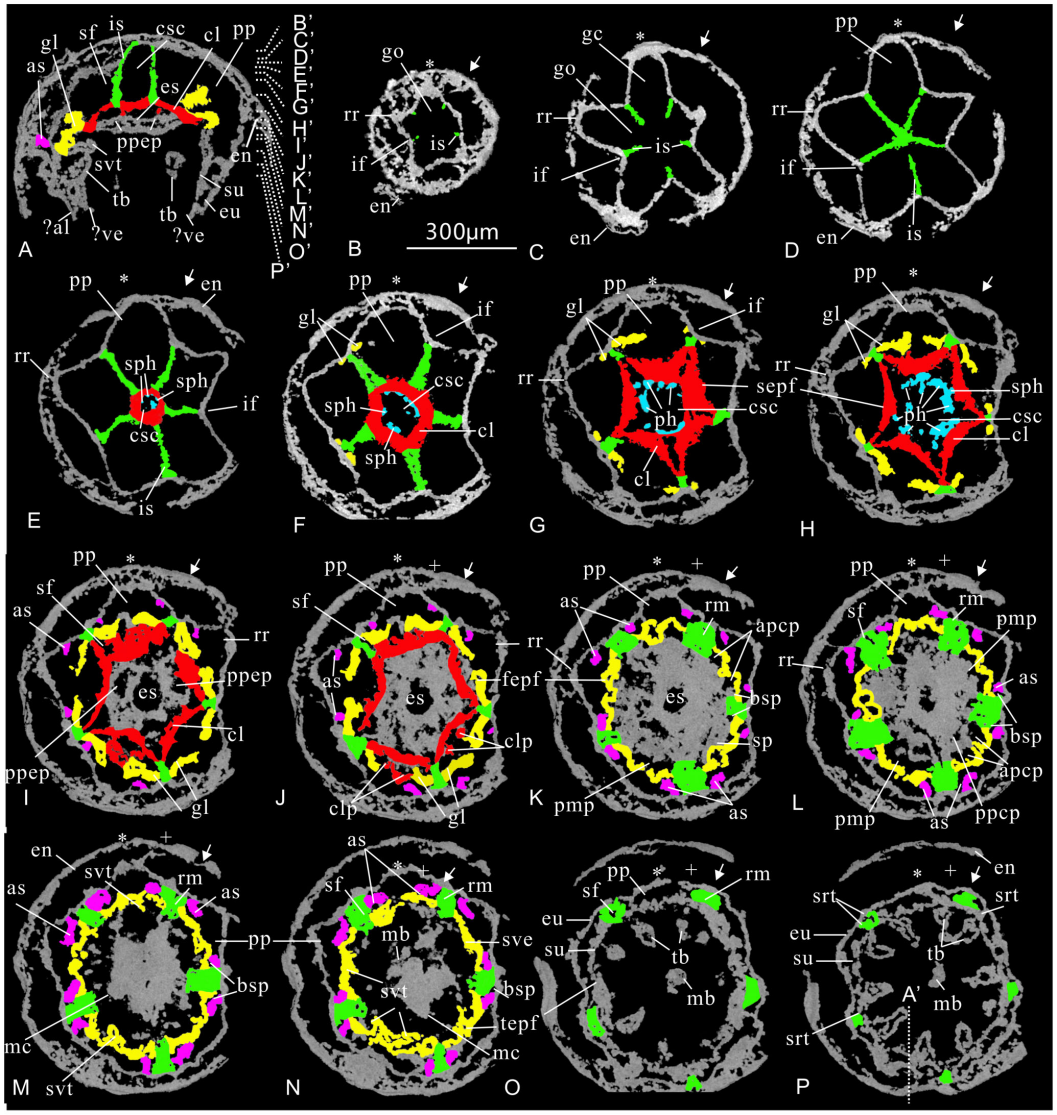
Interpretation of the virtual sections of cubozoan specimen ELISN31-5 from the Lower Cambrian Kuanchuanpu Formation, South China. The horizontal position of these virtual sections is marked as those in Figure 3. All sections share the scale bar (equals to 300 µm) seen in B.

Micro-CT reveals that the specimen ELISN31-5 preserves unequivocal internal anatomical structures (Figure 3). The specimen is composed mainly of two layers, respectively the outer exumbrella (eu) and inner subumbrella (su). The spacious gastric cavity between the exumbrella and inner subumbrella is constructed of a set of lamellae and gastric pockets. As the internal structures of the fossil change greatly at different levels, we illustrate and describe the fossil by successive virtual cross sections from the aboral to oral regions, as shown in Figures 3–4.

At the aboral stomach region, the cross section orthogonal to oral-aboral axis resembles a five-pointed star. Five points of the star, corresponding to five bulging, but sharp radial ridges of the apical part of the exumbrella in the perradii (marked ‘*’), intercalated with five depressed radial furrows (Figure 2B, Figure 4B–C). These five radial ridges become weaker and gradually disappear at the mid-level of the specimen (Figure 4I–J). At each incurved corner of the five-pointed star, five vertical, short and strikingly thin plate-like walls radiate from the adaxial side of the exumbrellar radial furrows. These straight plates, ca.15 µm in thickness, are interpreted as the septa. Because the septa in living tetra-radial medusozoans are located in the interradii (marked by a ‘→’) without exception, thus, we term them as interradial septa (is) (Figure 4A–B), which further provide the basis to define the orientation of the pentaradial fossils in addition to the oral-aboral axial. The interradial septa extend toward the body axis at the floor of the stomach cavity (Figure 4C) and then coalesce at the center at a lower level (Movie S1), subdividing the single gastric cavity into five broad, uniform, and nearly rhombic perradial pockets (pp) (Figure 4D), whcih can communicate with each other by gastric ostia (go) at the stomach floor (Movie S2). More orally, the central mass of jointed septa is developed into a small central stomach cavity (csc) (Figure 4E). This stomach cavity is greatly expanded in lower successive sections when each interradial septum is longitudinally cleft into two opposite vertical valves, thus forming five sharply arched, deltoid claustra (cl) in the perradii, a strip of tissue consisting of a double layer of endoderm connecting the interradial septa when seen in living staurozoans and cubozoans [3]. The claustra become sharper and more crescent-like in following successive levels (Figure 4G–H). At the same level, a short and tiny stalk is outgrown from the mid-point of each inner side of the claustrum at each perradius (Figure 4G–H). Each stalk gives rise to two rows of large phacellae (gastric filaments) in the perradii (Figure 4E–H). In more oral sections, these phacellae become slightly elongated, up to 75 µm in length (Figure 4F–H).

Internal to the external five-star-shaped body wall, the coalescence of the five claustra generates an inner, larger five-pointed-star framework at the outer margin enclosing an innermost starfish-like central stomach cavity (Figure 4F–I; Movie S1) that becomes wider and more spacious at the middle level of the specimen. This large space then narrows as the circular wall of the esophagus contacts the distal ends of the phacellae, which cause the central stomach cavity to separate into five abaxial interradial septal funnels (sf) and five adaxial perradial peri-esophageal pockets (ppep). The inner side of the claustra constructs the first endodermic pentagonal funnel (fepf) around the esophagus at this level (Figure 4I). The esophageal lumen, ca. 80 µm at maximal diameter, diminishes and disappears as it approaches the oral disc (Figure 4M and Movie S2).

The role of the claustra in constructing this embryonic medusa becomes more important as many other lamellae arise and are finally spliced with the claustra. The sheet-like gonad-lamellae germinate bilaterally from either side of the conjunction point of the septal roots and exumbrella at the rise of the claustra (Figure 4F–I). Thus the five interradial pairs of gonad-lamellae are equivalent to the typical gonads that occur in the exogonial pockets of living cubomedusae ([3], Figure 4). Each gonad-lamella continues to extend and points toward the central space of the perradial pockets; further orally, the distal ends of adjacent gonad-lamellae in the same perradial pockets are fused with a pair of the closest claustral projections (clp) that sprouted outwards from the mid-point of each claustrum at the adaxial side (Figure 4K–J), thus forming five pairs of ‘eyeglasses’ spectacle frame-like adradial peri-claustral pockets (apcp) (Figure 4K), five perradial peri-claustral pockets (ppcp) (Figure 4L), and a second zigzag-shaped endodermic pentagonal funnel (sepb) with its zigzag borders around the claustrum pentagon (Figure 4K–L; Movie S1). Adjacent adradial peri-claustral pockets are separated by a suspensorium (sp) at each perradius (Figure 4K), which is a bracket structure connecting the subumbrella and manubrium in extant cubozoans [17]. The suspensorium comprise two parts, which is easily recognizable in cross section as two triangles tip to tip (Figures 1E, 4K). Every two adradial peri-claustral pockets and one perradial peri-claustral pockets soon fuse together into a larger perradial mesogonial pocket (pmp) just below the oral disc (Figure 4L).

At the levels below the aperture, five pairs of short vertical walls quite close to the interradial septa, interpretable as accessory septa (as), emerge from one-fourth the length from the interradial furrow (if) to the perradial ridges of the exumbrella (Figure 4K–L). These accessory septa direct to the central space of the reduced perradial pockets in a manner much like the first appearance of gonad-lamellae. Externally, the individual accessory septum can also be recognized at the exumbrella as an inconspicuous shallow accessory radial furrow on either side of the interradial furrow. More orally, the distal ends of the accessory septa join with adjacent gonad-lamella and the thickened primary interradial septum, respectively, thus forming five pairs of tiny basal septal pockets (Figure 4K–L); this coalescence constitutes the third endodermic pentagonal funnel (tepf) with roughly straight borders around the level of the oral disc (Figure 4M–N). All of these three aforementioned pentagonal funnels are supported by the interradial septa whereas the first and second funnels are additionally connected by the perradial suspensoria.

Slightly below the oral disc, the first pentagonal funnel is free from the second pentagonal funnel as the perradial suspensoria and the primary interradial septa are completely detached (Figure 4L–M). As a result, the first pentagonal funnel and the esophagus met and finally coalesced, developing into the manubrium, whilst the second and third funnels fused. The third funnel finally develops into the endodermis of the vertical subumbrellar wall (Figure 4M–O). The exumbrella and subumbrella are connected and fixed by the interradial septa except along the medusa rim (Movie S2). Both the manubrial base and the uppermost subumbrella are pentagonal in cross section (Figure 4M–N).

Viewed in the longitudinal section, one side of the medusa rim is terminated by two, tiny less-distinct but sharp projections, possibly representing the apertural lappet and the velarium (Figure 4A and Movie S2). In addition to this possible velarium, there is a circular tissue bearing five pairs of perradial projections at one-third the height of the subumbrellar wall (Figure 4A,N; Movie S2), interpreted here as the secondary velarium (sve) with secondary velarial teeth. Probably, the function of both types of velaria is helpful to contract and to reinforce the bell during swimming.

Internally, the secondary velarium and other portion of subumbrella are connected to the exumbrella by the interradial septa, which appear as a thickened independent mass with a septal funnel in between the contiguous perradial pockets. These radial pockets, originally rhombic in shape in the apical part, are reduced into a smaller deltoid funnel (Figure 4I) and finally remain as a circle of marginal crescent spaces as the new pockets arise stepwise (Figure 4I–O). These crescentic spaces still exist between the exumbrella and subumbrella at the oral unit of the medusa. The thickened interradial septa, which probably contain retractile muscles (rm), become less distinct with the emergence of the five pairs of sub-interradial and hollow tentacular buds that arise from the subumbrella disposed just above the septal funnel (Figure 2P; Movie S1,S3). The lumen of the each tentacle bud communicates with the perradial pockets of the medusa.

In sum, the gastrovascular system of the embryonic medusa contains a suite of lamellae including the endodermic interradial septa, accessory septa, gonad-lamellae, and the esophageal wall, as well as their derived valves. These lamellae partition the whole gastric cavity into 45 pockets, which can be subdivided into seven units: five perradial pockets (pp), perradial peri-claustral pockets (ppcp), perradial mesogonial pockets (pmp), interradial septal funnel (sf), perradial peri-esophageal pockets (ppep) as well as five pairs of adradial peri-claustral pockets (apcp) and basal septal pockets (bsp). Due to the rise, separation and recombination of various endodermic lamellae, the profile of the medusa varies at different levels: starting firstly as a five-pointed star at the aboral end, it transforms into a pentagon in the middle part and finally become an almost equilateral decagon as it approaches the bell margin (Figure 4C–N).

### The internal anatomy in specimen ELISN108-343

SEM observation of specimen ELISN108-343 reveals that this hemispherical fossil embryo, ca. 620 µm in maximal width and 450 µm high, is almost entirely exposed except for a small preserved piece of egg envelope (Figures 2C,D, 4). Differing from ELISN31-5, there are five uniform prominent large triangular lobated perradial apertural lappets (pal) evenly distributed at the bell margin, ca. 60 µm high, pointing toward the body axis. Additionally, five pairs of small adradial apertural lappets (aal), ca. 30 µm in height, are situated in between the larger ones. All these lappets are interiorly solid with a thickened rim that is continuous with adjacent ones, thus constituting a complete thickened medusa rim. A wide circular velarium, a diagnostic feature of living cubozoans ([17], Figures 16,28), ca. 60 µm wide, is seen closely adjacent to the subumbrellar rim (Figure 2C). A central robust manubrial primordium with a still closed mouth is surrounded by a whorl of five pairs of interradial subumbrellar tentacles at the same level around the velarium. Thus, the bell aperture is not the real esophageal lumen but represents the opening of the subumbrellar cavity. Externally, besides the relatively deep coronal, middle and sub-apical circumferential grooves, three or four shallow horizontal circumferential grooves can also be recognized from the analysis of the 3D Micro-CT reconstruction images (Movie S6); all of these ring grooves are interrupted by the interradial furrows that correspond to the interradial septa (Figure 2D).

**Figure 5:**
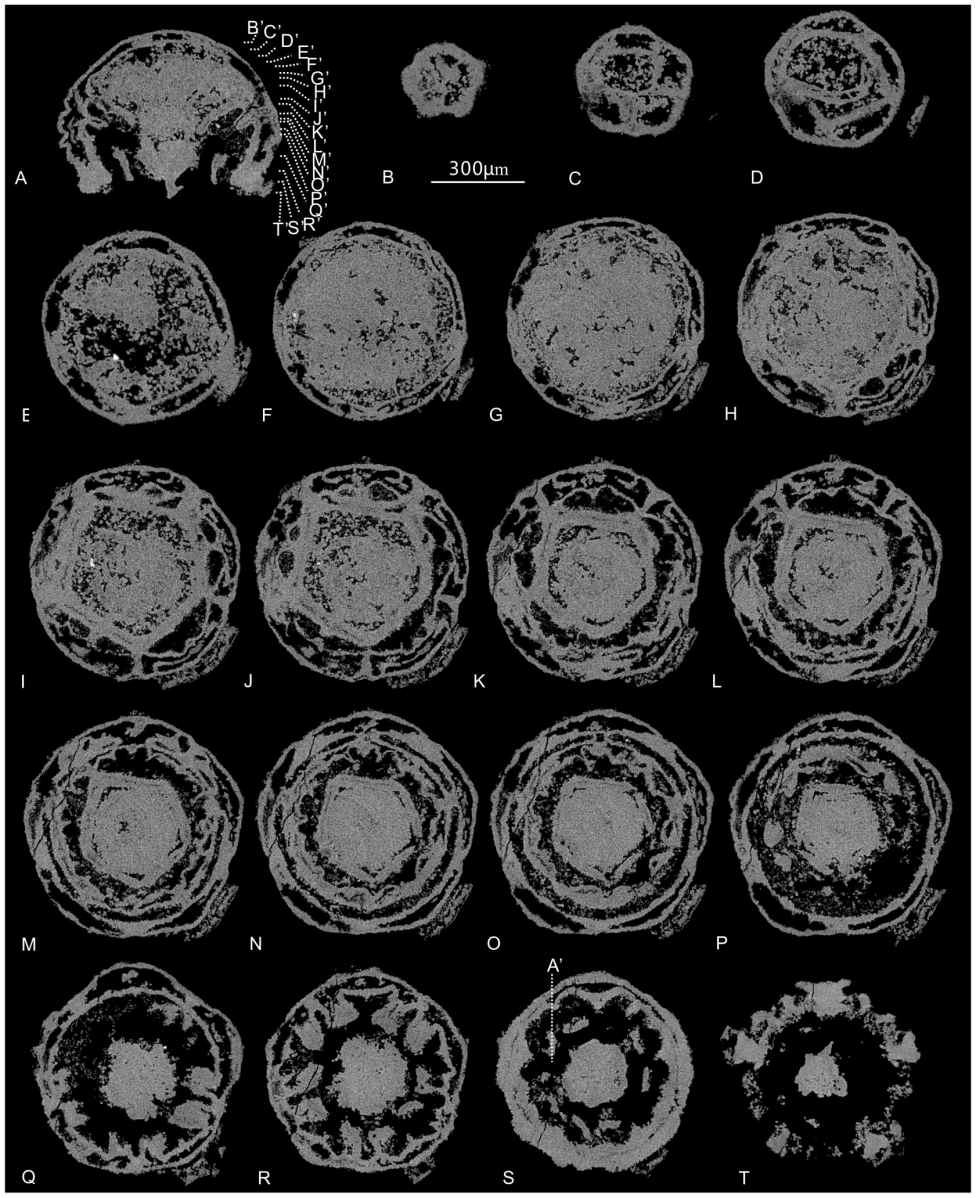
Micro-CT photographs of the microscopic cubozoan (ELISN108-343) from the Lower Cambrian Kuanchuanpu Formation, Shaanxi, South China. (A) Virtual vertical section marked with A′ in (S) by a vertical dotted line. (B–T) Successive virtual transverse sections through the orthogonal to oral-aboral axis starting from the aboral end, with their horizontal levels marked by B′–T′ with white dotted lines. Scale bar = 300 µm.

Micro-CT reveals that the constrution of the spacious gastric cavity between the exumbrella and subumbrella of the medusa-shaped specimen ELISN108-343 is more complex than that of ELISN31-5, as seen in successive virtual sections from the aboral to oral region (Figures 5).

**Figure 6:**
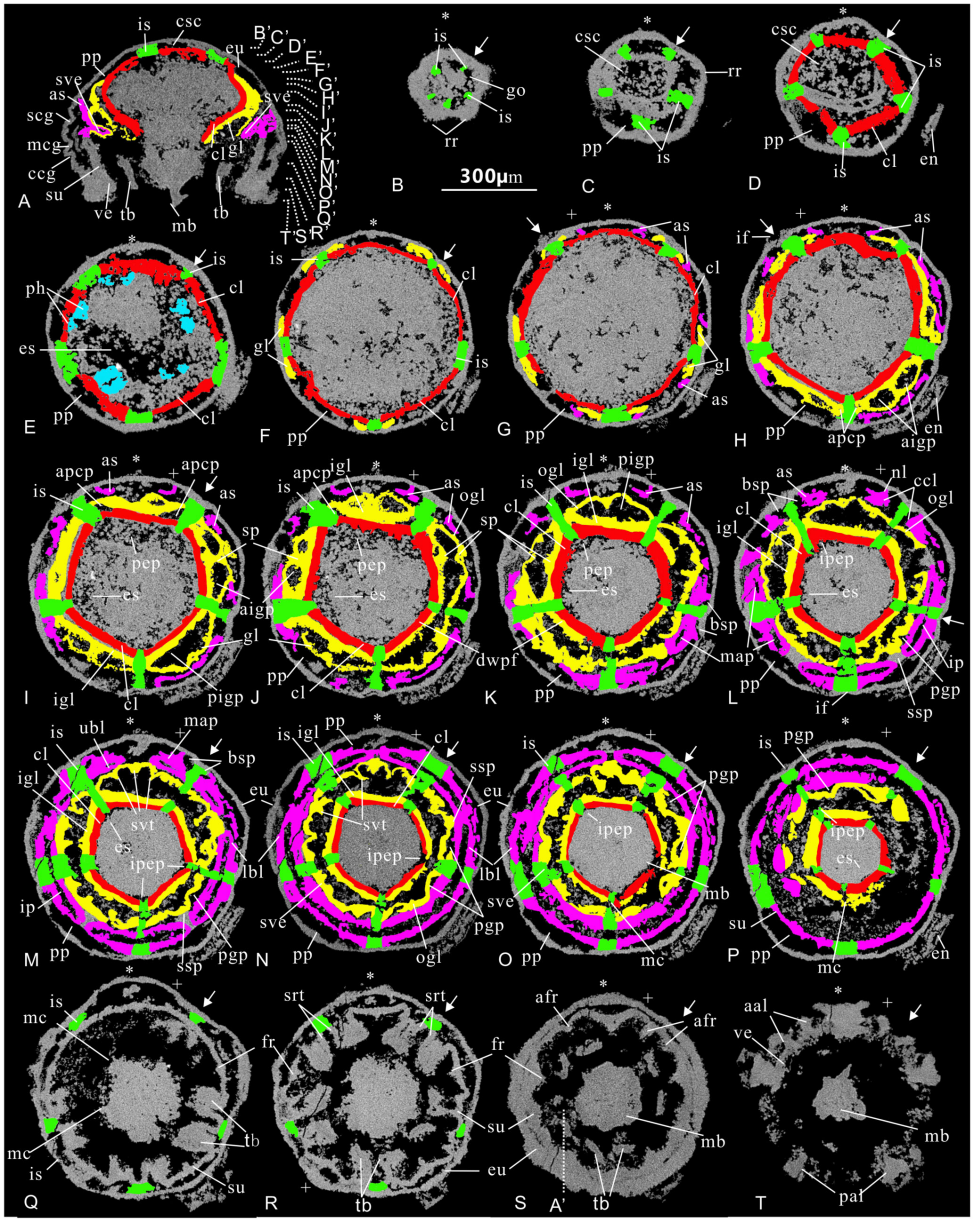
Interpretation of the virtual sections of the cubozoan specimen ELISN108-343 from the Lower Cambrian Kuanchuanpu Formation, Ningqiang, Shaanxi, South China. The position of these virtual sections is indicated as those in Figure 5. Scale bar = 300 µm.

Although the aboral part of the embryonic medusa is hemispherical, the apex in cross sections is a round-cornered pentagon containing five narrow perradial pockets delimited by five short interradial septa (Figure 6B). The claustra, derived from the central fusion and the lateral expansion of the interradial septa (Figure 6B), are closer to the apex and exumbrella than those in ELISN31-5. At the aboral region, the space of the perradial pockets is rather more restricted than those in ELISN31-5 versus a large central stomach cavity. As in ELISN31-5, one pair of large phacellae (gastric filaments) arise from the middle point of each claustrum (Figure 6E), projecting into the central stomach cavity.

In the middle region, the central stomach cavity (Figure 6B–C) and the surrounding perradial pockets are well partitioned into many small pockets near the level at the middle circumferential groove (mcg), where the embryonic medusa reaches its maximal width. The gonad-lamellae and the adradial septa in the perradial pockets are also situated quite close to the medusa apex (Figure 6F–H). Each gonad-lamella is further subdivided (Figure 6F–H) into an abaxial outer gonad-lamella (ogl) and an adaxial inner gonad-lamella (igl); more orally, both the inner and the outer gonad-lamellae extend and fuse together at each perradius where they are connected by a perradial suspensorium (sp); this connection leads to the formation of five pairs of adradial intra-gonad pockets (aigp) in horizontal section resembling ‘eyeglasses’ spectacle-frames (Figure 6G and Movie S4). Notably, the inner gonad-lamellae stand tightly close to and parallel with the claustra, and in between the claustra and inner gonad-lamellae, there are five pairs of narrow adradial peri-claustral pockets (apcp), as those in ELISN31-5. However, as seen in successive virtual sections toward the oral end, these pockets are highly reduced in size, appearing as five straight inconspicuous slits between the inner gonad-lamellae and claustra (Figure 6H–T; Movie S4).

The combination of the inner gonad-lamellae and claustra forms a vertical double-walled pentagonal funnel connecting the exumbrella by the interradial septa which in cross sections look like a long nail with its basal end sticking to the exumbrella, thus constructing a most important and strong framework or mainstay to give supports to all other derived components of the medusa. This double-walled pentagonal funnel tapers orally and embraces the basal part of the manubrium (Figure 6A). Unlike the perradial peri-esophageal pockets in ELISN31-5, five interradial peri-esophageal pockets (ipcp) surround the esophagus peripherally in ELISN108-343 (Movie S4). The column-like esophagus with quite limited central lumen, hangs freely from the double-walled pentagonal funnel and extending into the subumbrellar cavity.

In orally successive sections, each pair of independent adradial intra-gonad pockets in the vicinity soon merges into as one larger perradial intra-gonad pocket (pigp) as each suspensorium divides into two opposite miniature triangular parts (Figure 6G; Movie S4). Similar structures are visible in both ELISN31-5 and living cubozoans, such as *Charybdea xaymacana* ([17], Figure 10) and *Tripedalia cystophoa* ([17], Figure 23). Notably, the suspensorium in fossil and extant cubozoans is situated in between different endodermic lamellae, perhaps representing a functional convergence for improving efficiency in contractile swimming of the medusa.

The shape of each pair of sub-interradial accessory septa varies orally at successive levels. At the same level as the root of the manubrium, the accessory septa make their appearance looking like a simple band oblique to the subumbrellar wall (Figure 6G); and slightly orally, they appear to be a recurved walking-stick-shaped wall in cross section with the short proximal end standing vertically to the exumbrellar wall and the long distal end almost perpendicular to the adjacent interradial septum (Figure 6H–J). More orally, the accessory septa gradually transform into a structure with four diverticulae in virtual cross sections reminiscent of a peacock head with gapping beaks (Figure 6J–L). The proximal end of each accessory septum and the short lamella at the cockscomb of the “peacock head”, whcih are located closer to the adjacent interradial septum, termed here as the “neck-lamella (nl)” and “cockscomb-lamella (ccl)”, respectively. Two lamellae at the position of the beak, which are branched from the long distal end of the accessory septa, are dubbed accordingly as an adaxial “upper beak-lamella (ubl)” and a “lower beak-lamella” (lbl) abaxial to the body axis. In the levels close to the apical circumferential groove, the neck-lamella and cockscomb-lamella construct a tiny basal septal pockets (bsp) by bridging with adjacent interradial septa (Figure 6L). Each upper beak-lamella (ubl) and the outer gonad-lamella constitute an adradial peri-gonad pocket (pgp), which further fuses with the adjacent peri-gonad pocket into an interradial peri-gonad pockets (Figure 6L) as the interradial septa break off at the specific point between the inner and outer gonad-lamellae. The relatively thick upper beak-lamellae form five pairs of marginal adradial pockets (map) with the outer adjacent lower beak-lamellae. Slightly more orally, two contiguous marginal adradial pockets merge into a larger interradial pocket (ip) (Figure 6L). Further orally, neighboring interradial peri-gonad pockets and interradial pockets are separated in the perradii by five secondary suspensoria (ssp), which connect the outer gonad-lamellae, upper and lower beak-lamellae (Figure 6M). Each part of the secondary suspensorium is constituted by the coalescence of a primary suspensorium (sp) and the two distal ends of the accessory septa within the same perradial pocket (Figure 6M–N; Movie S4). Both the interradial and perradial pockets are crescent-shaped at the level of the middle circumferential groove (mcg)(Figure 6M), and this imparts to the exumbrella an oral round-cornered pentagon. Furthermore, orally, both the space of the interradial and perradial pockets becomes diminished.

The secondary velarium is also a double-walled ring-like structure extending from the deep subumbrella and projecting inward and upward slightly lower than the apical circumferential groove (Movie S5). It is substantially made up of the upper beak-lamellae of the accessory septa and the outer gonad lamellae banded to subumbrellar wall by both the interradial septa and secondary suspensoria (Figure 6M–N). The distal part of the secondary velarium is free from the subumbrellar wall as the fusion of interradial pockets (Figure 6A, M–P; Movie S4).

The apertural velarium, which is bound to the subumbrellar wall, is suspended perpendicularly by five larger solid bracket-like frenula triangular in cross section in the perradii and ten smaller adradial frenula (afr); all of these frenula are responsible for reinforcing the velarium as in living forms [18]. Especially, the lower ends of the frenula on the velarium, either perradial or adradial ones, are consistent well with the adjacent apertural lappets in shape and disposition. The lumen of these frenula directly communicates with the upper perradial pockets, being strikingly different from the secondary velarium in histological derivation.

Histologically, the endoderm of the subumbrellar wall is continuous with the lower beak-lamellae of the accessory septa (Movie S5). At the level of the middle circumferential groove, five interradial pairs of hollow tentacular buds arise from the deep level of the subumbrellar wall. Each tentacular bud comprises two ‘roots’ and a big massive ‘head’, as seen in cross sections; specifically, the adjacent roots of the paired tentacles (srt) appear to be directly rooted at the interradial septa (Figure 6Q,R; Movie S4). Thus, each tentacle is a composite product derived histologically from both the endodermis of the exumbrella and subumbrella; the paired tentacle roots still serve the function of the septa connecting both the umbrellae toward the medusa margin. Septal roots of the tentacles are also present in ELISN31-5 (Figure 4O). The tripartite tentacle has a saddle-shaped base, a capitate middle part and an oblate top; the basal part is perpendicular to the subumbrellar wall and directs to the manubrium whereas the oblate top is parallel to the subumbrellar wall. Below the level of the septal roots, the perradial pockets can communicate with each other. The external coronal circumferential groove of the bell, equal to the level of the septal roots, differentiates the bell margin with the velarium and the apertural lappets from the other portion of the bell, and the body wall of both umbrellae thickens orally. As a result, the space of the perradial pockets is greatly reduced and resembles a circle of many discontinuous arched slits (Figure 6S).

The central stomach cavity and esophageal cavity are filled with solid material (Figure 6A, E–T; Movie S5), interpreted here as unconsumed yolk grains, a case consistent with the closed and immature status of the functional mouth.

In summary, in the gastrovascular system of the specimen ELISN108-343, there are up to 70 gastric pockets, which can be subdivided into nine units: five perradial, interradial, perradial intra-gonad, interradial peri-esophageal pockets, as well as five pairs of adradial peri-claustral, adradial intra-gonad, basal septal, adradial peri-gonad and marginal adradial pockets.

### Further specimens showing internal anatomies

Many other specimens in our collections also show five pairs of tentacles. However, it is difficult to identify their internal anatomy because the gastric cavities are almost completely occupied by homogeneous calcium phosphate, probably representing a large bulk of unconsumed yolk.

**Figure 7:**
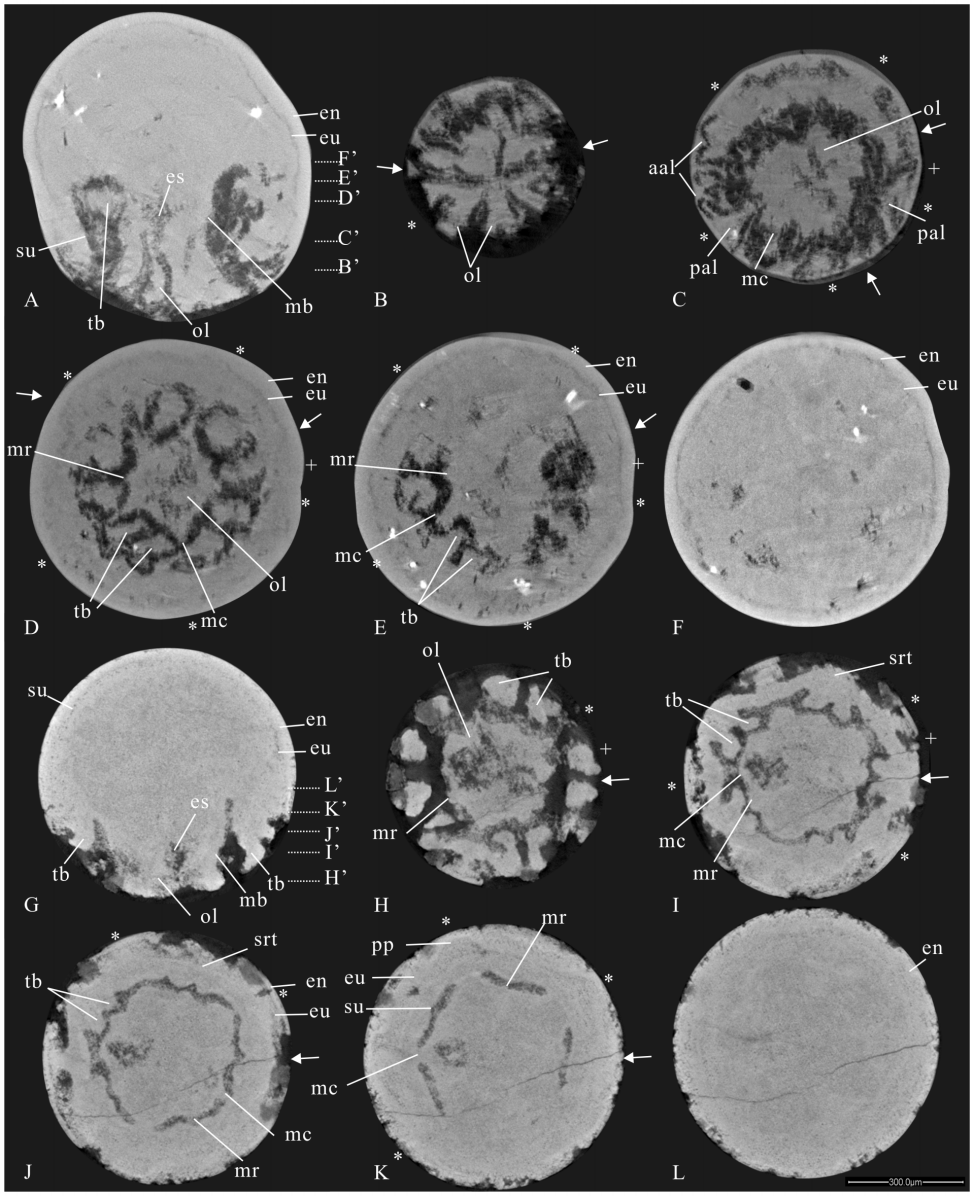
Micro-CT sections of two cubozoan specimens with five pairs of tentacles. (A–F) ELISN 96-103. (A) Vertical section along the oral-aboral axis. (B–F) Horizontal sections. (G–L) ELISN66-15. (G) Vertical section along body axis. (H–L) Horizontal sections. Scale bar = 300 µm.

Specimen ELISN96-103 is an ellipsoid embryo with quite a thick egg envelope showing limited exposure of the oral side (Figures 2E, 7A; Movies S7, S8, S9). The specimen bears five kite-shaped sharp perradial oral lips that point centripetally, with their distal ends meeting at the manubrial center (Figure 7B–C). Micro-CT reveals that the tissue of the egg envelope is whiter and brighter in color than enclosed soft-tissue. A five-cornered elongate and stout flask-shaped manubrium, about half the height of the medusa, is surrounded by five interradial pairs of fairly evenly spaced capitate tentacles deep at the subumbrellar wall, five large triangular perradial apertural lappets and five pairs of small adradial apertural lappets (Figure 7C–E). The lumen at the aboral end of the manubrium clearly represents the real esophagus. The bulk of the gastric cavity as well as the esophagus beneath the oral disc are filled with egg yolks (Figure 7A,F). The oral lips not only occur at the mouth opening but extend aborally deep into the esophagus around the level of the oral disc. Specifically, the flaring manubrium with nearly a opened mouth, exhibits five small triangular interradial longitudinal ridges intercalated with five long but sharp perradial manubrial corners extending outward up to the subumbrellar wall between the adjacent pairs of tentacular buds (Figure 7D and Movie S7). Clearly, these perradial corners differ in disposition from the interradial manubrial corners in specimens ELISN31-5 and ELISN108-343 but do resemble the suspensoria (or perradial mesenteries) in living cubozoans (Figure 1, see also [19], [20]). Besides the coronal circumferential groove, there are several other continuous horizontal circumferential grooves (Figure 2E–F), which are not interrupted by the interradial septa as in the specimen ELISN108-343.

Specimen ELISN66-15 is globular in external shape, with a cone-shaped manubrium terminated by an expanded mouth (Movie S10). Its height is less than half of the medusa. In cross section, the basal part of the manubrium appears as a stout pentagon with perradial manubrial corners connected with the subumbrellar wall (Figure 7J–K; Movie S11), similar to the disposition in specimen ELISN96-103. Each side of the manubrial pentagon is parallel with those of the pentagon-shaped subumbrellar and exumbrellar walls (Figure 7K). In the middle part, these pentagonal manubrial corners become sharper and are intercalated with five small triangular interradial longitudinal ridges that become longer near the summit of the manubrium; the top of manubrium seen in cross section thus resembles a pentagon with ten corners (Figure 7H).

The oral lips in specimen ELISN66-15 are also situated in the perradii. Similar to the scenario in specimen ELISN108-343, each pair of shallow subumbrellar tentacular buds share two fused interradial septal roots (Figure 7I). Specifically, the aboral surface of these tentacular buds exhibits a shallow longitudinal groove (tg) near their distal ends. The velarium is wide and extends inward and is surrounded by five triangular but stout apertural lappets at each perradius (Movies S10, S12). Unlike ELISN108-343 and ELISN96-103, no adradial apertural lappets are present. Despite the background of yolk materials, the triangular perradial pockets are still discernible in between the umbrellae at the level of the manubrial stalk (Figure 7J–K). From the aboral end onward, the bulging radial ridges of both umbrellar pentagons switches from the perradii to the interradii due to the thickening of the body wall and the rise of the tentacles in the interradii (Figure 7H–I).

Specimens ELISN66-14, ELISN25-79, and ELISN35-42 are enclosed within globular envelopes and are much more similar to ELISN66-15 than to others (Movies S13, S14), Their tentacular buds are capitate with oblate distal ends parallel to the subumbrella as those in ELISN108-343. These tentacles are located quite close to the bell margin. In particular, all of these specimens bear five perradial apertural lappets and exhibit shallow grooves on the aboral side of the tentacular bud whereas their manubria show different widths (Figure 2G–I). Remarkably, the shallow groove on the aboral side of the tentacles has no counterpart in extant cubozoans as far as we know. Some species of extant Scyphomedusae have a similar food groove but on the adradial side of the tentacles [21].

## Discussion

### Morphological comparisons of the figured fossils

Although all of these figured pentaradiate fossils exhibit five paired tentacular buds arising from the subumbrellar wall, and probably represent a new order-level monophyletic group, they can not be assigned to same genus or species. The specimen ELISN108-343 is apparently different from ELISN31-5 in many aspects, such as external morphology and internal anatomy, especially the organization of gastric pockets and the derivation of subumbrellar endoderm. In ELISN108-343, the endoderm of the subumbrella is continuous with the upper part of accessory septa, whereas in ELISN31-5, it is mainly derived from the upper part of gonad-lamellae. ELISN108-343 is closer to other specimens in the presence of the perradial or adradial apertural lappets and elongate, stout pentagonial manubrium; however, as discussed above, there are some other minor differences among them, such as the disposition of the mouth corners and the morphology of tentacles. The occurrence of tentacles at the different levels of the bell cavity and the shape of the manubrium, which is cone-shaped in ELISN108-343 and ELISN66-15 but trumpet-shaped in ELISN96-103, possibly falls within the scope of the ontogenetic variations.

Much similarity appears when the specimen ELISN108-343 is compared with GMPKU3089, for both of their apertures bear five concentric pentaradially arranged stout perradial apertural lappets (principle rays in [13], fig. 3c) and ten smaller adradial apertural lappets (paired subordinate intercalary rays in [13], fig. 3c) around the aperture. Furthermore, the internal anatomy of the “inner wall”, “outer wall”, “radial wall”, “paired radial lobes”, “recurved wall”, “polygonal axial structure” and “interradial ridge” in specimen GMPKU3089 [13] correspond respectively to the subumbrella, exumbrella, interradial septum, paired tentacular buds, accessory septum, manubrium, frenulum in the specimen ELISN108-343 (Figure S1). The upper abradial ridges in GMPKU3089 are interpretable as secondary velaria. The claustrum, the lower part of the inner wall in GMPKU3089 (fig. 3l in [13]), is also present in both fossils. The manubrial corner in both specimens is situated in the interradii (Figure S1) and the apparently constricted bell opening indicates the presence of a velarium. However, there are some evident differences between them. (1) Each frenulum in specimen GMPKU3089 seemingly has a radial canal, which is unseen in ELISN108-343. (2) The radial depressions in GMPKU3089 are slightly different from the ‘eyeglasses’ spectacle frame-like adradial peri-claustral pockets. (3) The claustra in ELISN108-343 are closely attached to the inner gonad-lamella (Fig. 4I–P). (4) The ridges on the claustra in GMPKU3089 (fig. 3i in [13]) correspond to the adradial inner part of suspensorium in ELISN108-343 (Figure S1). (5) The double-walled funnel constituted by the claustra and inner gonad-lamellae around the basal part of the manubrium in ELISN108-343 is unseen in GMPKU3089. Although both of their manubrial mouths remain closed, indicating a prehatched embryonic status, these two specimens most likely represent two different taxa rather than developmental variations.

Th figured fossils, including GMPKU3089, might not be *Olivooides multisulcatus* or embryos of *Punctatus emeiensis*. 1) The embryo of *P. emeiensis* is jar-shaped with a high cap-shaped small aperture [22], [23]; 2) the integument (or theca) of the embryo bears diagnostic stellate ornaments in many developmental stages; 3) all figured fossils are naked devoid of any theca and the stellate ornaments in the very late embryonic stage; 4) the theca, although originally organic in biological composition, is a kind of exoskeleton more resistant to decay during the taphonomy and diagenesis than soft-tissue. In the Kuanchuanpu Formation, the stellate theca and fertilization envelope are usually preserved [11], [13]; in contrast, the soft-tissue of *P. emeiensis* is quite rare and hitherto has only been found in one specimen (GMPKU3087 in [13]) with no detailed internal anatomy available. Thus, if fossilized embryos preserved detailed internal anatomy of soft tissues and originally the epidermis of the soft-tissue secreted an external theca but both of these are enclosed by the outermost fertilization envelope, the theca would have had high potential to be preserved.

Although the known fossils vary greatly in their internal anatomy, it is still inadequate to establish new taxa systematically at generic level, when considering the relative scarcity of such fossils with preserved internal anatomy and the morphological variations possible in different developmental stages, as well as various taphonomic factors.

### Systematic affinities

#### Echinoderm affinity

Pentameric symmetry is a form of symmetry well-known in echinoderms which are well defined as a group with a stereomic calcareous endoskeleton composed of articulated ossicles, a water vascular system with tube feet, and a straight or coiled gut between mouth and anus. Ontogenetically, it is well supported that the pentamerism of adult echinoderms is metamorphosed from a bilaterian larva by twisting of the body around the oral-aboral axis and their vascular system is derived from the left hydrocoel only [24]. The fossil record of echinoderms coincides with ontognetic observations on living forms. Ancestral echinoderms and their stem groups in Cambrian Stage 3 are bilateral or asymmetrical [25], [26]; their descendants first acquired tri-radial and pentaradial symmetry around Cambrian Stage 4 or 5 [27], [28]. Hitherto no echinoderm fossils are known from the Cambrian Terreneuvian [27].

As convincingly disputed in Dong et al. [13], these pentaradiate fossils from the Kuanchuanpu Formation,are definitely not echinoderms especially because these specimens usually possess a flexible organic exoskeleton rather than a calcareous endoskeleton, a water-vascular system without tube feet, and no sign of an anus following the gut. Additionally, the presence of various gastric lamellae within the body cavity, including the septa, paired gonad-lamellae and paired tentacles in the interradii, and the suspensoria, frenula and appertural lappets in the perradii, as well as an annular velarium, provide additional but fundamental evidence to strengthen this argument.

Nontheless, as the case in figured fossils, the direct development of embryos without a feeding larval stage is common among different clades of echinoderms. And in an extreme case, if these embryonic fossils were echinoderms, the calcium carbonate spicules would have been initiated at this state of development and probably could not be seen in a fossil; the water-vascular system comes later without tube feet, all of which will add to the difficult task of setting their systematic classifications. In such a case, the body cavity can provide clues to diferentiate those of cnidarians from echinoderms. The radial symmetric gastrovascular cavity of cnidarians is the only cavity with a single opening which serves as both mouth and anus. Whereas incipient echinoderm larvae, which are usually bilaterally symmetric externally, have several independent cavities including a digestive system and several pairs of coeloms ([24], Figure 28-4).

Some kinds of echinoderm larvae, especially the echinopluteus larvae of sea urchins, the mesogens of starfish, and the vitellaria larvae of brittle stars, resemble the current fossils in morphology given that the ciliary bands, an important diagnostic characteristic of echinoderm larvae, can hardly be fossilized. It is necessary here to further distinguish the current fossils from these echinoderm larvae.

If the mouth and anus were not identified, superficially the echinopluteus larvae resemble the Chinese fossils in conical shape and four or five pairs of elongate arms. However, the echinopluteus larvae have bilateral symmetry in shape through their 2-, 4-, 6-, 8-, or 10-armed ontogenetic stages [29]. The arm pairs in a typical echinopluteus larva, which are similar to paired tentacles in the figured fossils, are not equal in length. It is more discernible when the arms are pierced by many calcite spines. In some species, the echinopluteus larvae have a large pre-oral lobe [29] similar to the medusozoan manubrium. However, the pre-oral lobes are oblate in shape whereas the medusozoan manubrium is polygonal in virtual cross section. Especially in cubozoans, the cone- or trumpet-shaped manubrium is connected to the subumbrellar wall by four or five perradial suspensoria. Secondly, the distal end of the pre-oral lobes will not further develop a mouth with radial lobes as the manubrium of medusozoans. Thirdly, the pre-oral lobe, although surrounded by arms, is not located at the oral center.

In several brooding species of starfish with direct development, the late-stage pelagic mesogens resemble somewhat the non-stellate theca of *Olivooides* in the hemispherical dome-like oral region and an aboral region with pentamerous radial bulges intercalated with five radial grooves [30]. The aboral and oral regions are separated by a deep equatorial groove from which protrudes with some podia. The podia are the strinking feature to make distinguish the mesogens from the figured fossils.

Pentaradial symmetry is evident in the vitellaria larvae of ophiuroids, and the mouth of the larvae is beset with five triangular juvenile skeletal plates and some buccal tube feet [31], a pattern quite similar to the apertural lappets seen in the specimens ELISN108-343 and GMPKU3089. However, the vitellaria larvae has a quite large pre-oral lobe, which is quite distinct from current fossils.

#### Cnidarian affinity

Apparently, the features of penta-radial symmetry and a polygonal manubrium with radial lobes in the figured fossils completely exclude an affinity with bilateral anthozoans but are compatible with medusozoans (Figure 1). The Hydromedusae have a typical velum without frenula and velarial canals connected to the gastric cavity [2]; their septa, if present, are highly reduced without phacelli at the inner end; the tentacles of Cubomedusae are solid; in addition, the claustra and apertural lappets are never reported in this group.

The staurozoans are a group of stalked, sessile medusozoans characterized by eight or four pairs of adradial arms (anchors) each bearing a cluster of capitates secondary tentacles. This grop represents the earliest diverging clade of extant medusozoans based on recent phylogenetic analyses [32], and thus, many endodermic structures, such as interradial septum with paired gonads, phacellus (gastric filaments), and the claustrum, are seen in the same disposition in both staurozoans and cubozoans. However, besides the external topography, the specific disposition of gonads differs in these two orders. Notably, one family of staurozoans (Cleistocarpidae) [33] develops a claustrum, which is considered to be homologous to those in cubozoans [20]. The gonads in this family, when the claustra appear, develop within the mesogonial pockets rather than the exogonial pockets as in cubozoans [3] (Figure 1F–G).

Apparently, most of the figured fossils, except for ELISN31-5, develop five or ten centripetal triangular marginal lappets, which are absent in cubozoans. Specifically in ELISN108-343, the leaf-like gonad-lamellae develop in the exogonial pockets. The most striking feature in these fossil embryos is the paired tentacles, which are unseen in staurozoans (Figure 1F).

As noted above, a similar fusion of interradial septa forming four perradial pockets at the apical part of the specimen ELISN31-5 is widely seen in the stalk of extant cubozoans [20], [34]. Thus, concerning the basal position of cubozoans in the medusozoan phylogenetic tree, this indicates that the apical part of ELISN31-5 retains a more primitive condition than other contemporary and extant cubozoans. It also gives support to the proxy hypothesis [17] that the stalk and basal stomach of the staurozoans, delimited from other parts by the level of the interradial phacelli, which are considered homologous across all classes of medusozoans, are equivalent to the apical part in Cubomedusae. The difference between these two classes, probably including scyphozoans, as has been suggested [20], reflects their respective adaptation for sessile and swimming habits.

The current fossils resemble living scyphozoans in thier paired leaf-like gonads attached along the interradial septa. However, scyphozoans lack the typical claustrum and frenulum, as in both the current fossils and cubozoans (Figure 1H). Additionally, the feature of paired tentacles seen in all the figured specimens is also absent in living scyphozoans.

Several lines of evidence, including the specimen GMPKU3089, support the new fossils being ancestral cubozoans. (i) concentration of subumbrellar tentacles in the interradii is only present in cubozoans; (ii) the characteristics of the claustrum in the described materials exclude their affinities to the Anthozoa, Hydrozoa, as well as Scyphozoa, but suggest more in common with Cubozoa (Figure 1E, G); (iii) the morphology and disposition of the gonad-lamellae agree well with those of cubozoans and scyphozoans (Figure 1E,H) [3]; (iv) specifically, the features of the suspensorium and a velarium supported by the frenula, occur exclusively in extant cubozoans (Figure 1B–D); (v) the lack of rhopaloids, which are seen in all living medusozoans but are absent in these fossils, possibly is either related to their embryonic status or is a preservational bias; (vi) living cubozoans have only two kinds of pockets separated by claustra: exogonial and mesogonial [3] (Figure 1E). Apparently, so many new gastric pockets in the current fossils are derived from further partitioning of the exogonial pockets and mesogonial pockets because new lamellae arise. (vii) The process of fusion and separation of these gastric lamellae and pockets, such as the fusion of the septa and the separation of suspensoria in the current fossils, is identical to that in modern cubozoans [17] (Figure 1E).

In sum, all of the noted features in the figured fossils agree closely with those of the extant cubozoans. However, some of the fossil forms are more complicated and are more highly specialized than extant cubozoans, which are generally noted for more complex behavior than other cnidarian classes [35] and are recognized as a “highpoint” in the development and evolution of cnidarians [36]. The analysis of the internal anatomy of these Cambrian fossil cubozoans undoubtedly offers invaluable implications for understanding the evolution of characteristics in cubozoans and the early radiations of medusozoans.

### Further discussion on characters in fossil cubozoans

#### Paired subumbrellar tentacular buds

The most striking feature of these cubozoan embryos is the paired tentacular buds superficially, as mentioned above, sprouting from the subumbrella but histologically derived from both umbrellae (Figures 2Q, 3Q–R). Generally, adult cubozoans have wide pedalia and contractile tentacles located around the medusa rim (Fig. 1A–B). However, in living cubozoans and scyphozoans, the tentacles originate ontogenetically from the subumbrella [17], [37], [38] and the pedalia do not develop in the youngest stage of cubozoans [20], [39]; also in living cubozoans, the velarium, tentacles and rhopaloids consist of subumbrella epidermis plus gastrodermis [2]. It is also worth considering that the subumbrellar tentacles are present in the sea anemone-like cnidarian *Eolympia* [10] and an unidentified fossil cnidarian polyp [9] from the same horizon and locality. All of which leads us to propose that subumbrellar tentacles probably represent a rather primitive condition at least in cubozoans.

Living cubozoans include two monophyletic groups: chirodropids and carybdeids [1], [40]. Chirodropids have multiple tentacles attached to each pedalium, whereas carybdeids always have a single tentacle per pedalium [1], and one bell corner of the medusa margin with two tentacles is also seen in *Tripedalia binata* [41]. The tentacles of fossil cubozoans, including those from Utah and Mazon Creek [8], exhibit a similar pattern to the carybdeids. Notably, all of the tentacles in the current embryos are rooted in or closely related to the interradial septa, a configuration directly comparable to those of living cubozoans [17]. Thus, compared with the tentacles, the interradial septa in cubozoans and the current embryos are fundamental components in the construction of the medusae, and this is supported by the fact that the primary septa appear first during an early ontogenetic stage of both anthozoans and medusozoans.

#### Septa and their derivatives

The new microscopic fossils unprecedentedly preserve a delicate and complicated framework that have only be seen in living cubozoans, including interradial coalescent septa with claustra, septal funnels, gonad-lamellae, suspensoria and frenula, velaria, as well as many pockets. The endodermic lamellae, additionally including the secondary suspensorium (ssp), the endodermic subumbrellar wall, velaria, and secondary velaria, are directly or indirectly derived from the interradial septa and the accessory septa. Thus the septa and their derivatives play a fundamental role both in the organization and classification of these embryonic cubozoan fossils, and analysis o this can provide new insights into the evolutionary history of medusozoans.

**Figure 8:**
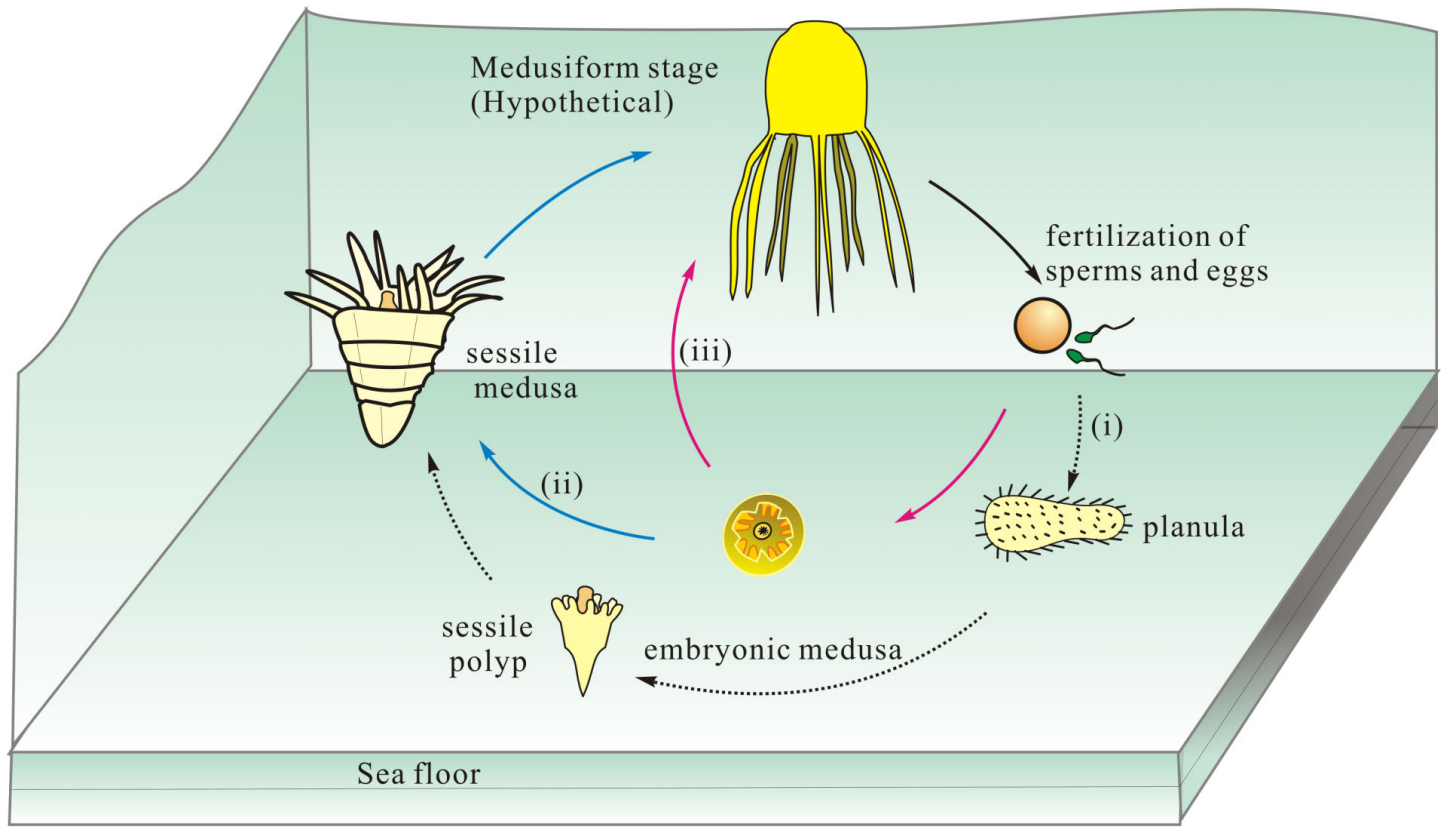
Hypothetical life cycle of Cambrian cubozoans. (i) A typical biphasic mode with planula and polyps. (ii) A sessile medusa stage after hatching of embryos. (iii) A shortened life history without free living planula and sessile polyp.

The claustrum, a vertical tissue composed of two endodermic layers separated by a thin mesoglea, is destined to develop later than the primary septa [33] (Figure 7A–G; [34], Figures 6–11). Nevertheless, in combination with the primary septum, the claustra in our fossil cubozoans do play a fundamental role in helping to determine their systematic positions.

In modern medusozoans, the paired gonads are usually located on both lateral sides of the primary interradial septa [3]. Specifically in the cubomedusae and scyphomedusae, the gonads have developed into a pair of leaf-like lamellae attached along the edge of the interradial septum within the mesogonial pocket ([3], Figure 3; [2], Figure 167A–B). The gonad in the cubomedusae, which contains numerous sperm or many ovaries, is covered by two endodermic layers separated by a thin mesoglea [20]. Thus in histology, it can be regarded as an endodermic lamella capable of producing gametes. With regard to the prehatched conditions of these fossils, the gonads usually appear ontogenetically very late in larger individuals of living cubomedusae [20], but these microscopic fossils were unable to produce gametes from the incipient buds of the ‘gonads’ at the embryonic stage. In addition, in living cubomedusae, the gonads have free distal margins that never fuse with the adjacent lamellae of the same perradial pockets even if in some individuals they develop so much as to overlap each other in the perradii [20]. The gonad-lamellae in the new fossil cubozoans also exhibit free distal margins near the aboral region; but more orally, they go on to produce several branches that finally combine with adjacent lamellae, thus demonstrating a high potential for flexibility in a manner similar to the adjacent septa and accessory septa. Functionally, this plays a very important role in constructing the vascular system of the bell, which is not restricted to the function of yielding gametes. In one words, thus it is more logical to name this structure as a ‘gonad-lamella’ rather than ‘gonad’ or ‘incipient gonad’.

In mature female individuals of these fossils, the gonad-lamellae are presumed highly likely to have produced large eggs that would have developed into the fossil embryos preserved. Notably, the eggs or embryos in the gonad-lamellae of extant cubozoans with internal fertilization are enclosed in a layer of gelatine [17], which is dissoved before the embryos are released from the manubrium. We interpret the egg envelope of the current embryos as this gelatinous layer. However, it is still not clear whether the accessory septa in these fossils are capable of producing eggs.

The appearance of the accessory septa in the adradii of these fossils is exceptional. In side view of some extant cubozoans, there are eight longitudinal adradial corner pillars and adradial furrows on the either side of interradial septa (Figure 1B), but no accessory septa develop. In other extant medusozoans as far as we know, all of their septa and other endodermic lamellae including gonad-lamella are exclusively directly derived from four primary interradial septa and no new septa are directly produced from the exumbrellar wall. However, a similar scenario is commonly seen in anthozoans, such as rugosozoans and hexacorallians, in which many new septa are supplemented the adjacent existing septa (or mesenteries) in a sequential or cyclic manner and in various patterns in the body wall [42], [43] (Figure 1I). Such new septa have indirect temporal and spatial correlations with the four primary septa. One of the major differences between these two classes is that the septa in anthozoans are always simple-sheet- or plate-like (Figure 1I) whilst those in medusozoans exhibit a greater ability and flexibility capable of, and potential for bifurcation, differentiation and combination, which in turn have suppressed the further addition of new septa from the exumbrella. Thus, the anthozoans and medusozoans reflect two fundamentally different strategies to partition the gastric cavity so as to improve the efficiency of digestion and to enhance the stiffness of the increased body size.

Thus an evident pattern shared by fossil and modern cubozoans is emerging that new endodermic elements in the body wall are always added peripherally or are attached to the existing lamellae. In addition, all these gastric lamellae are inevitably allocated to either of the two systems clutched by the suspensoria: the central system of the manubrium and the peripheral system of the medusa bell.

#### Radial ridges and apertural lappets

In the fossil cubozoans, especially ELISN31-5, the consistency between internal anatomy and external ridges or grooves is evident. Here two different patterns are discerned for the aboral and oral unit of the medusa. In the aboral part, the perradial pockets appear as bulged radial ridges whereas interradial septa appear as a set of more or less concaved radial grooves or furrows. This pattern is well illustrated on the stalk of staurozoans [34] as well as in some anthozoans [2]. In ELISN108-343 and in living cubozoans this pattern is clearly restricted to the apex, and the oral part fits in with another pattern because there, the body wall, especially the mesoglea in the interradii is somewhat thickened and bulging outwardly ready to accommodate the sprouting of tentacles. However, these interradial ridges can be distinguished from the perradial ridges by the presence of interradial furrows and perradial apertural lappets. The latter pattern is also visible in ELISN31-5, ELISN108-343 and living cubozoans [16].

Most living cubomedusae do not develop the perradial and adradial apertural lappets as do these Cambrian fossil cubozoans. But similar triangular perradial lappets at the bell rim are present in *Chiropsella bart* (Chirodropida) ([16], fig. 3A). The apertural lappets are alternatively somewhat comparable to velarial canals in juvenile living cubozoans (Figure 1C–D). These velarial canals, eight in the adradii and four pairs on the side of the perradial frenulum, are primarily triangular in shape in quite young specimens [20]. As the organism grows, the velarial canals will branch dichotomously within the velarium of the full-grown medusa ([17], [20], Figures 74–78). However, the velarium and the velarial canals, which are connected with the perradial pockets, are substantially derived from the subumbrella; in contrast, the solid apertural lappets in these fossils are derived from the exumbrella and they are not homologous structures.

### Development mode and life cycle

It is well known that the development mode of cnidarians depends mainly on the richness of the egg yolks [44]–[46]. Most scyphozoans, with their eggs generally smaller than 300 µm, exhibit an ephyra stage in their life history that normally precedes the medusa and earlier sexual planula stages [45], [46]; and some rare particular forms have lost the polypoid stage or exhibit a holopelagic life cycle with continuous development from the fertilized large yolky eggs to medusae without any sessile stage by producing rather large eggs (ca. 300 µm in diameter).

The life history of the Cubomedusae is not as well known as that of Scyphomedusae. Limited data known on cubozoan eggs or embryos are ranging from ca. 50 µm to 200 µm in diameter [35], [36], [47], [48]. Typically, the cubozoans eggs are set free from the gonads, leaving the exogonial pockets and to enter the central stomach cavity through the gastric ostia. They get fertilized in the female gastric cavity and finally are liberated from the manubrium forming motile planulae [15], [35]. The planulae soon settle down on their apical part and transform into creeping [49] or sessile primary cubopolyps each with four primary perradial tentacles; in the later stages the polyp entirely converts into a single swimming cubomedusa, a specific metamorphosis different from the typical strobilation of the scyphozoans [39]. Direct development in extant cubozoans has not been reported, as far as we know. However, the planula, probably including the primary cubopolyp of *Morbakka virulenta* (Kishinouye, 1910) (Charybdeida, Cubozoa) is found metamorphosed into a stalky cubopolyp before hatching from the egg envelope, which is ca. 100 µm in diameter [48]. Thus, it means the planulae of the cubozoans are not always necessary for development outside the egg envelope and the life history among different species of Cubomedusae appears more flexible than ever thought. And so the direct development of cubozoans without a cubopolyp stage is not impossible if the cubozoan eggs contain much more yolk.

All of the available fossil embryos are ca. 450–600 µm in diameter, thus indicating that the eggs have sufficient yolky nutrients to sustain their lecithotrophic development. The complicated construction of the gastrovascular system of the medusa, the manubrial primordium with closed mouth opening, and especially the associated egg envelopes of the new fossils clearly demonstrate their very late pre-hatched embryonic stage. Their youngest adult forms are estimated to have been at least 6 mm in width concerning the accommodated space of the radial pockets that housed the gonad-lamellae and the large eggs.

Based on these extraordinary but limited Kuanchuanpu specimens, it is still difficult to determine whether these fossil embryonic cubozoans would grow up in post-embryonic stages as (i) sessile polyps, like the life history of extant cubozoans, (ii) sessile medusae, or (iii) pelagic medusae after hatching (Figure 8). Presumption (i) seems unlikely considering the evident differences in topography and internal anatomy between these figured embryonic fossils and extant primary cubopolyps. The extant primary cubopolyp of *Tripedalia* lacks gastric septa, gastric pockets and septal funnels, but bears a circle of primary tentacles in the perradii surrounding a closed functional mouth cone [39], [50]. The primary tentacles will transform into the perradial rhopaloids and later the interradial medusa tentacles appear before the radial polyp metamorphosis into a medusa with its various internal apparatuses, i.e. septa, claustra, and paired gonads, arranged in tetraradial symmetry [39]. Thus, it tends to support the hypothesis that the current fossil embryos are most likely equivalent to immature cubomedusae rather than cubopolyps in general morphology and detailed anatomical structures. Thus, the ontogenetic development of these fossils did not undergone a typical free living planula and a polypoid stage as in extant cubozoans [39].

We prefer the third scenario (iii) that indicates a short-cut holopelagic life cycle (Figure 8). The hatched juvenile individuals grow up directly into a larger pelagic medusa, which later produces sperms or large eggs with sufficient yolks from the gonad-lamellae. The fertilized large eggs might directly hatch into a medusa. However, the alternative sessile medusa mode (ii) cannot be excluded if the hatched individuals settled on the substrate with their aboral blunt end; this sessile medusa would grow up along the oral-aboral axis as the middle coronal grooves are continuously duplicated in a manner similar to that of *Punctatus emeiensis* [11] (see also in ELISN108-343; Movie S6); and then the sessile medusae would perform asexual reproduction [2] or leave the substrate starting their pelagic life (iii).

### Evolution of symmetric patterns

Besides typical tetramerous radial symmetry, some medusozoans seemingly have a genetically fixed pentaradial pattern rather than one derived from developmental malformation [51], [52]. Apparently, all of the external and internal components of the embryos described here are evenly arranged in a strict pentaradial pattern. Thus, pentaradial symmetry is no longer exclusively a diagnostic characteristic of crown-group echinoderms, as previously suggested [12], [13], [53]. This recognition provides a solutions to the problematic situation of diverse pentamerous fossils such as *Punctatus emeiensis* and its relatives [12] if the morphology of their exoskeleton is mirrored with the soft-tissues. It further implies that ancestral medusozoans most likely had a wide spectrum of radial symmetry. Anabaritids, an indicative and diverse group of tubular fossils with triradially symmetrical exoskeletons found during the Ediacaran-Cambrian transition period, are also probably stem group cnidarians [12], [54].

In conclusion, these Early Cambrian embryos from South China, although still in a pre-hatched stage and revealing pentaradial symmetry, can be identified as the earliest-known ancestral cubozoans based on the extraordinary phosphatization of their internal anatomy which exhibits high fidelity to the original biological structures. The pentaradial symmetry is no longer exclusively a characteristic of crown-group echinoderms; it was also possessed by a diversity of Early Cambrian cubozoans from the Kuanchuanpu Formation. With regard to their external morphology and internal structures, these cubozoan embryos are closer to the cubozoan medusae rather than to cubopolyps, thus providing strong evidence of animals undergoing direct development without the free-living planula and polypoid stages typically seen in extant cubozoans. The Cambrian fossil cubozoans are more complicated than extant forms due to the evolutionary invention of new endodermic lamellae which exhibit frequent fusion and separation.

## Supporting Information

Figure S1 **Drawings and reinterpretation of virtual sections of an** ***Olivooides*** **-like embryo (GMPKU3089)** [**13**] **.** A–D, respectively redrawings of fig. 3f, 3j,3k, 3l, respectively in [13].
(TIF)

Movie S1 **Micro-CT movie of succesive transverse sections of ELISN31-5 from the Kuanchuanopu Formation, South China.**
(MP4)

Movie S2 **ELISN31-5, succesive lateral sections of ELISN31-5.**
(MP4)

Movie S3 **Micro-CT movie of external profile of ELISN66-15.**
(MP4)

Movie S4 **Micro-CT movie of transverse sections of ELISN108-343.**
(MP4)

Movie S5 **Micro-CT movie of lateral sections of ELISN 108-343.**
(MP4)

Movie S6 **Micro-CT movie of external profile of ELISN108-343.**
(MP4)

Movie S7 **Micro-CT movie of transverse sections of ELISN96-103.**
(MP4)

Movie S8 **Micro-CT movie of lateral sections of ELISN96-103.**
(MPG)

Movie S9 **Micro-CT movie of external profile of ELISN96-103.**
(AVI)

Movie S10 **Micro-CT movie of external profile of ELISN66-15.**
(MPG)

Movie S11 **Micro-CT movie of transverse sections of ELISN66-15.**
(MP4)

Movie S12 **Micro-CT movie of lateral sections of ELISN66-15.**
(MP4)

Movie S13 **Micro-CT movie of transverse sections of ELISN66-14.**
(MP4)

Movie S14 **Micro-CT movie of external profile of ELISN66-14.**
(MPG)
