# Supplementary figures and images for: Early Cambrian Pentamerous Cubozoan Embryos from South China
Source: PLoS One. 2013 Aug 12;8(8):e70741. doi: 10.1371/journal.pone.0070741 (PMC3741300; doi:10.1371/journal.pone.0070741)

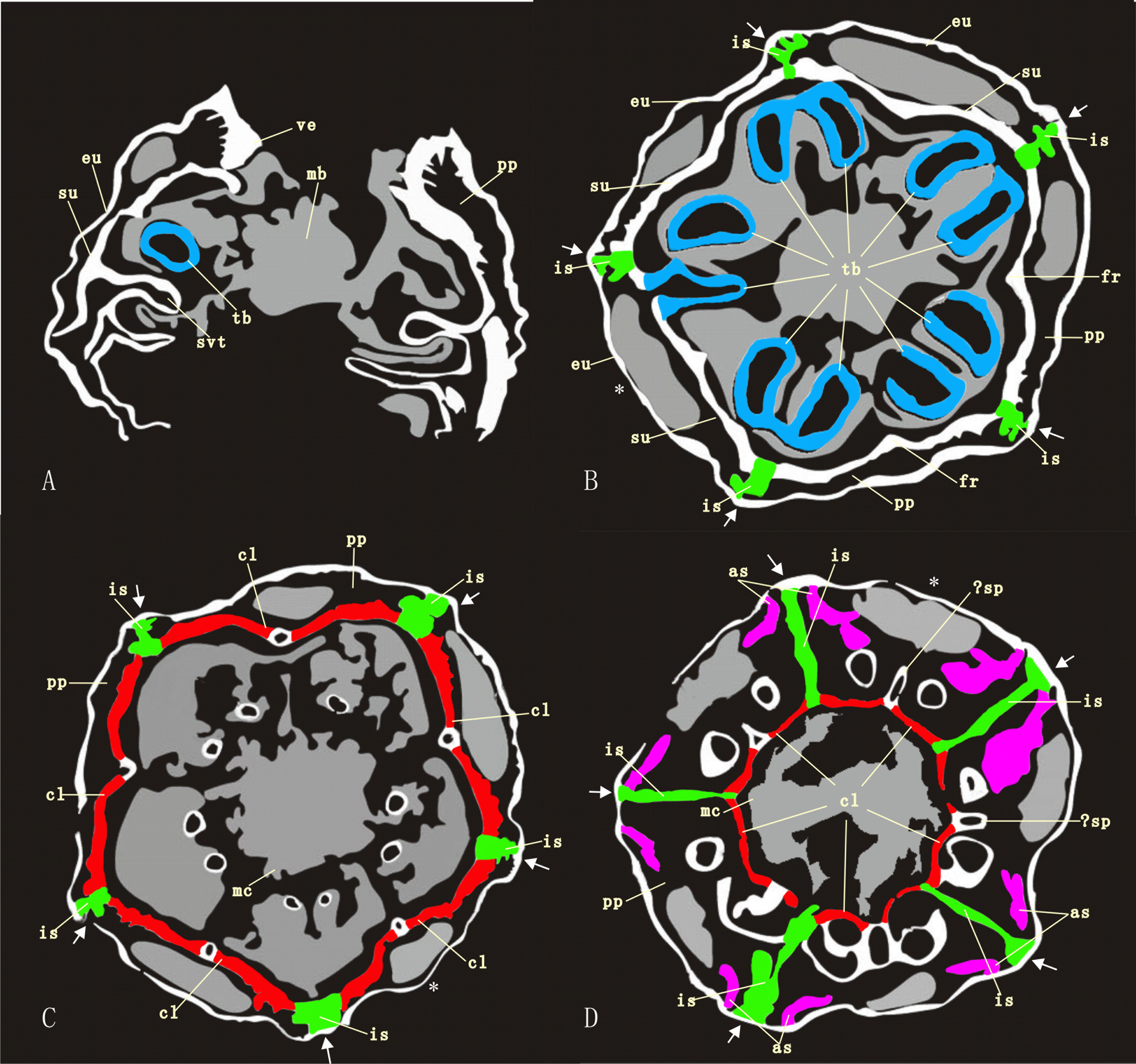

Supplement: Figure S1 — Drawings and reinterpretation of virtual sections of an Olivooides -like embryo (GMPKU3089) [13] . A–D, respectively redrawings of fig. 3f, 3j,3k, 3l, respectively in [13]. (TIF) [file pone.0070741.s001.tif]
